# Low-dose intranasal deferoxamine modulates memory, neuroinflammation, and the neuronal transcriptome in the streptozotocin rodent model of Alzheimer’s disease

**DOI:** 10.3389/fnins.2024.1528374

**Published:** 2025-01-13

**Authors:** Jared M. Fine, Jacob Kosyakovsky, Tate T. Bowe, Katherine A. Faltesek, Benjamin M. Stroebel, Juan E. Abrahante, Michael R. Kelly, Elizabeth A. Thompson, Claire M. Westby, Kiley M. Robertson, William H. Frey, Leah R. Hanson

**Affiliations:** ^1^HealthPartners Institute, Neuroscience Research, HealthPartners Neuroscience Center, Saint Paul, MN, United States; ^2^Minnesota Supercomputing Institute, University of Minnesota, Minneapolis, MN, United States

**Keywords:** intranasal, deferoxamine, streptozotocin, Alzheimer’s disease, neuroinflammation, transcriptome

## Abstract

**Introduction:**

Intranasal (IN) deferoxamine (DFO) has emerged over the past decade as a promising therapeutic in preclinical experiments across neurodegenerative and neurovascular diseases. As an antioxidant iron chelator, its mechanisms are multimodal, involving the binding of brain iron and the consequent engagement of several pathways to counter pathogenesis across multiple diseases. We and other research groups have shown that IN DFO rescues cognitive impairment in several rodent models of Alzheimer Disease (AD).

**Methods:**

This study was designed to probe dosing regimens to inform future clinical trials, while exploring mechanisms within the intracerebroventricular (ICV) streptozotocin (STZ) model.

**Results:**

Five weeks of daily IN dosing of Long Evans rats with 15 μL of a 1% (0.3 mg), but not 0.1% (0.03 mg), solution of DFO rescued cognitive impairment caused by ICV STZ administration as assessed with the Morris Water Maze (MWM) test of spatial memory and learning. Furthermore, IN DFO modulated several aspects of the neuroinflammatory milieu of the ICV STZ model, which was assessed through a novel panel of brain cytokines and immunohistochemistry. Using RNA-sequencing and pathway analysis, STZ was shown to induce several pathways of cell death and neuroinflammation, and IN DFO engaged multiple transcriptomic pathways involved in hippocampal neuronal survival.

**Discussion:**

To our knowledge this study is the first to assess the transcriptomic pathways and mechanisms associated with either the ICV STZ model or DFO treatment, and the first to demonstrate efficacy at this low dose.

## Introduction

Intranasally administered deferoxamine (DFO) has promise as a novel treatment for neurodegenerative disease and neurological insult. Intranasal (IN) delivery allows drugs like DFO to bypass the blood–brain-barrier and be delivered to the brain extracellularly along the olfactory and trigeminal nerves within minutes (Thorne et al., 2004; Chen et al., 1998; Frey, 1997; Thorne et al., 1995). Intranasal delivery has the added benefits of minimizing systemic exposure thereby decreasing side-effects, as well as being non-invasive. Deferoxamine is an approved generic antioxidant and anti-inflammatory drug that binds iron with very high affinity but has limited brain penetrance with systematic administration (Di Paola et al., 2022). Free iron accumulates abnormally in the brains of individuals with Alzheimer’s disease (AD), Parkinson’s disease, and other brain disorders (Rao et al., 2022). In the brains of people with AD, free heme, which also contains iron, also increases and both the free heme and iron inactivate the human brain muscarinic acetylcholine receptor required for memory *in vitro* (Venters et al., 1997; Atamna and Frey, 2004; Fawcett et al., 2002). Intranasal DFO has been shown in animals to treat a variety of brain disorders in which iron accumulates abnormally and even to improve memory in normal and healthy mice (Fine et al., 2020). This is an example of repurposing an existing drug to treat PD, AD, stroke, and other brain disorders by using non-invasive IN delivery to bypass the blood–brain-barrier and target DFO to the brain. Interest in DFO as a potential treatment for neurodegenerative disease has piqued in light of the recent realization that ferroptosis, a form of regulatory cell death based on unregulated iron levels, is involved in the development of neurodegenerative diseases and neurological insult (Stockwell, 2022). Ferroptosis in response to increased iron levels leads to lipid peroxidation, reactive oxygen species (ROS) production, mitochondrial dysfunction and neuroinflammatory responses resulting in cellular and neuronal damage (Tang et al., 2020; Jarrahi et al., 2020).

Several recent studies show that IN DFO is beneficial in animal models of neurodegenerative disease and neurological insult, and the mechanisms of neuroprotection are both disease-specific and non-disease specific. Intranasal DFO has been shown to reduce functional deficits in both genetic and surgical rodent models of Alzheimer’s disease including a tau model (Fine et al., 2012), APP/PS1 models (Hanson L.R. et al., 2012; Fine et al., 2015; Guo et al., 2013), and the intracerebroventricular streptozotocin model (Fine et al., 2017). Intranasal DFO improved functional recovery in several rat models of Parkinson’s disease including the 6-hydroxydopamine surgical model (Fine et al., 2014), and the a-synuclein rAAV model (Febbraro et al., 2013). Intranasal DFO significantly decreased the lesion size in an ischemic stroke model in rats, and resulted in functional improvement with both pre-and post-treatments (Hanson et al., 2009). Deferoxamine is also being tested in clinical trials as a potential treatment for intracerebral hemorrhage (ICH) and subarachnoid hemorrhage (SAH) (Selim et al., 2019; Foster et al., 2022), though administration in these trials is not intranasal. Mechanisms of neuroprotection in the aforementioned models include disease-specific changes in Alzheimer’s models including changes in processing of tau and amyloid (Hanson L. et al., 2012; Fine et al., 2012; Guo et al., 2013), while the Parkinson’s models included changes to processing of tyrosine hydroxylase and α-synuclein (Febbraro et al., 2013; Fine et al., 2014). Multifactorial mechanisms of non-disease specific neuroprotection shared across multiple disorders is summarized in a review by Kosyakovsky et al. (2021), and points toward changes in multiple cellular pathways including anti-inflammatory cascades, insulin signaling, HIF1α, and GSK3β.

To advance translation of IN DFO toward clinical trials, this study sought to find the lowest functional dose of IN DFO while further exploring novel mechanistic pathways of neuroprotection. A previous clinical trial of DFO-treatment in AD patients demonstrated some potential for benefit (Crapper McLachlan et al., 1991). However, this trial included daily intramuscular injections that lead to side-effects, which was the impetus to develop intranasally administered DFO in an attempt to minimize systemic exposure (Kruck et al., 1993). Previous studies have shown that a 10% solution (3 mg) of IN DFO reverses memory loss and oxidative stress in the ICV STZ rodent model of AD (Fine et al., 2017). This inducible model does not focus on either amyloid or tau pathology, but rather features of neurodegeneration and neuroinflammation (Grieb, 2016). To better characterize this model, the efficacy and mechanisms of lower doses of IN DFO were assessed using behavioral testing, multiplex analyses, immunohistochemistry (IHC), and RNA-sequencing. In addition to determining the lower dose limit of efficacy, this study expands upon previous studies with the first RNA-sequencing data for effects in the brain of the ICV-STZ model and DFO-treatment.

## Materials and methods

### Animal care

Studies were approved under HealthPartners Institute IACUC #17-097. Male Long-Evans rats were individually housed and given water and nutrients *ad libidum*. After 5 days of quarantine, rats were acclimated to handling for 5 days prior to surgery. Animals were checked daily for their duration on site, and twice daily for one-week post-surgery. Fluid and pain medications were given individually as needed at the discretion of the onsite veterinarian. Males were chosen for this initial dose-range finding study, with females to follow in a subsequent study. Female rats also need a different dose of ICV-STZ to induce a comparable model (Fine unpublished; Bao et al., 2017).

### Experimental design

**Figure 1:**
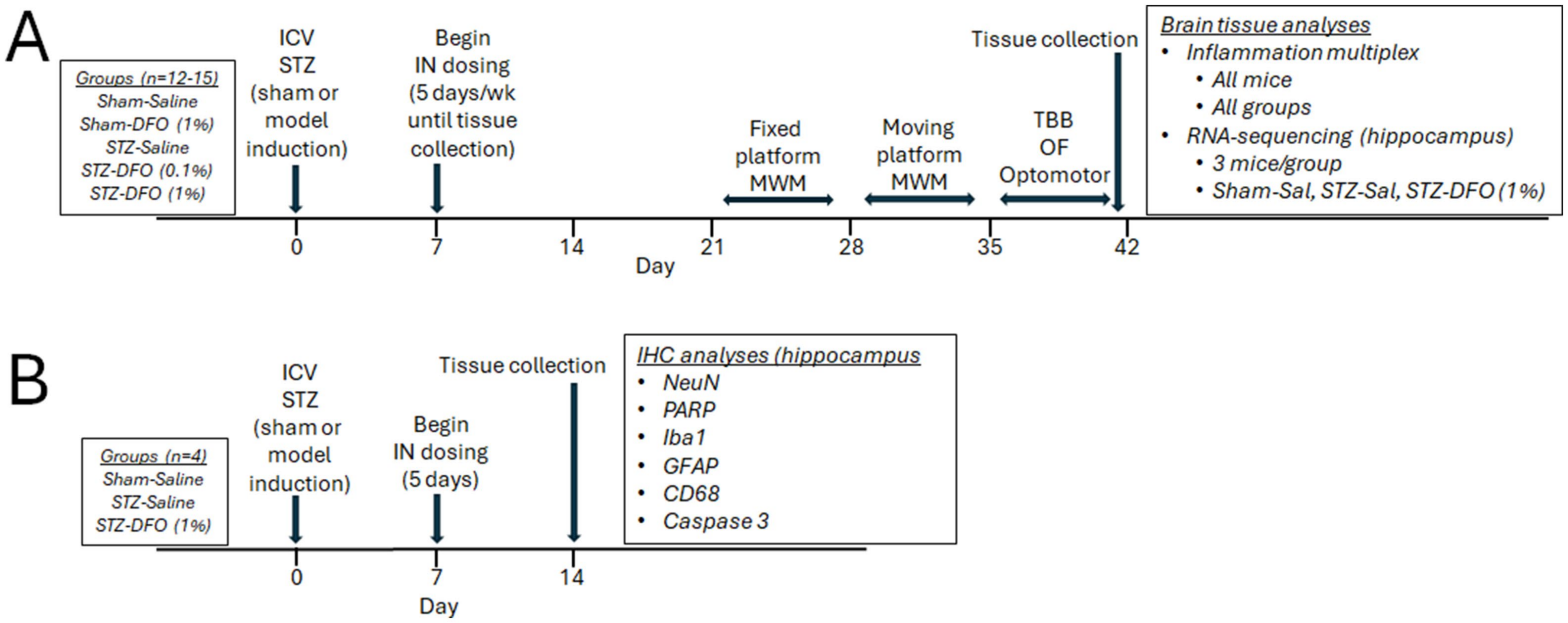
Visual representation of study design and timeline. **(A)** Five treatment groups dosed for 5 weeks and subjected to behavior tests followed by multiplex and RNA-sequencing. **(B)** Groups of four rats dosed for 1 week before immunohistochemistry. MWM, Morris Water Maze; ICV-STZ, intracerebroventricular streptozotocin; DFO, deferoxamine; IN, intranasal; TBB, tapered balance beam; OF, open field; Sal, Saline; IHC, immunohistochemistry.

Rats were randomized into five treatment groups of 12–15 rats. Treatment groups received stereotaxic surgery with an ICV injection of either citrate buffer (sham-model) or STZ (STZ-model). They also received IN delivery of either saline (control) or DFO at a 0.1% (0.03 mg) or 1% solution (0.3 mg). The five treatment groups included: (1) Sham-saline; (2) Sham-DFO 1%; (3) STZ-saline; (4) STZ-DFO 0.1%; and (5) STZ-DFO 1%. One week after surgery, rats started IN treatment with DFO or saline 5 days/week for 5 weeks. Week 3 included fixed-platform MWM testing, week 4 included moving-platform MWM testing, while week 5 included tapered-balance beam, open field, and optomotor tests before euthanasia and tissue collection. Behavior tests were performed before IN dosing for each rat on a single day. Brain tissues were snap-frozen in liquid nitrogen for analyses with multiplex and RNA-sequencing. Brains from all rats were used for multiplex analyses, while RNA-seq analyses were from a subset of 3 rats from sham-saline, STZ-saline, and STZ-DFO 1%. Additional rats were also assigned to treatment groups sham-saline, STZ-saline, and STZ-DFO 1% (4 rats/group) for IHC of brain tissues. A week after surgery, these rats were given 1 week of treatment with IN saline or DFO and then euthanized and fixed with formalin 14 days after surgery. These rats did not undergo behavioral testing. A visual representation of the study design and timeline can be seen in Figure 1.

### STZ model induction

Surgery was performed under isoflurane anesthesia at 4% for induction and 2–4% for maintenance (SurgiVet, Norwell, MA, USA). Rats were implanted with a double-guided cannula (Plastics One, Roanoke, VA, USA) and then given either STZ (S0130; Sigma; IN, USA) or the vehicle of 0.05 M citrate buffer (sham). Holes were drilled in the skull at A/P: −0.8, M/L: 1.5 (on both the left and right sides). The depth of the pre-made cannula was 3.2 mm from the top of the skull. STZ dosage was 4.5 mg/kg. For both the STZ and citrate buffer solution, four μL were administered to each hemisphere (8 μL total). 8 μL ICV injections were given on the day of surgery and again 2 days later via cannula. Bone wax was applied to the holes drilled into the skull after the second injection.

### Intranasal dosing

Rats were anesthetized using isoflurane gas and treated with a pressurized intranasal delivery device (Impel Neuropharma; Seattle, WA, USA). Saline or DFO solution (15 μL) was delivered to each nostril by inserting the tip of the device roughly 12 mm into the naris and depressing the plunger. Deferoxamine solutions were made by mixing 5 mL of 0.2× PBS with 500 mg anhydrous DFO (Frasenius-Kabi, Lake Zurich, IL, USA) under sterile conditions. Serial 1:10 dilutions were performed to obtain 1 and 0.1%, respectively. The total dose for the 1 and 0.1% DFO solutions were 0.3 and 0.03 mg, respectively.

### Morris water maze

Testing was conducted in a round, flat-bottomed, white plastic tub with a diameter of 180 cm at the water surface in a room with visual cues. Water was 23°C and colored with white nontoxic tempera paint. Four drop points were set. The platform was a clear plastic circle placed 1 cm underwater. For the fixed platform task, rats were allowed to swim up to 60 s or until they located the platform. Rats who failed to locate the platform within 60 s were placed upon it. All rats were then given 20 s to rest before being removed from the platform and placed in the tank for the next trial. In the fixed platform task, rats completed four trials per day for 4 days. The moving platform task followed the same protocol as the fixed platform task, except the platform was relocated to a different quadrant of the tank for each of the 4 days. Data was digitally recorded with an overhead camera.

### Tapered balance beam

The balance beam was 1.34 m in length, elevated 0.9 m above the ground, and decreased in width from the starting point (6.5 cm) to the end point (1.9 cm). Cushioning was placed on the floor below. Rats were placed at the wide end and encouraged to walk across and enter an escape box at the narrow end. The test consisted of a training day, during which the rat became accustomed to crossing the beam, followed the next day by testing. During testing, each rat performed three trials spending 20 s in the escape box between trials. Trials were digitally recorded from both sides and manually scored for footslips by blinded observers.

### Optomotor

Rats were placed on an elevated platform inside a cylinder with black and white lines alternating vertically with diameters of roughly 2 cm. The cylinder was spun at a rate of 2 rotations per minute for 3 min. A digital video recorder was placed above the cylinder for later review and blind scoring. A head turn was counted if the rat’s head followed the cylinder for at least 15° without stepping or moving its body. If a rat fell off the platform, the cylinder was stopped until the rat was placed back on the platform and the cylinder was spun for an additional 20 s. After 3 falls a rat was removed and considered non-compliant.

### Open field

The open field consisted of an 85 × 77 cm arena and monitored by an overhead digital video recorder. Each rat was placed individually within the arena for 5 min, and their exploratory behavior and velocity were analyzed with Ethovision tracking system (Noldus, Leesburg, VA, USA).

### Frozen tissue collection and protein extraction

Rats were anesthetized and transcardially perfused with 120 mL of cold saline. Blood was collected prior to starting the perfusion and serum separated. After perfusion the head was removed and the brain was extracted. The brain was dissected into cortex and hippocampus, snap frozen in liquid nitrogen, and stored at −70C until analysis.

### Fixed tissue collection

Rats were anesthetized and transcardially perfused with 120 mL of cold saline followed by 120 mL of 10% neutral buffered formalin. The brain was removed and placed in a formalin post-fix for 24 h followed by a sucrose gradient before being sectioned for cryo-slicing.

### Multiplex analyses

Inflammation was assessed with a commercially available multiplex panel of 27 inflammatory markers (cat. No. RECYTMAG-65K; MilliporeSigma; Burlington, MA, USA) and measured on a Luminex Magpix plate reader (Luminex, Austin, TX, USA). Markers included EGF, Eotaxin/CCL11, Fractalkine, G-CSF, GM-CSF, GRO/KC, IFN-γ, IL-1α, IL-1β, IL-2, IL-4, IL-5, IL-6, IL-10, IL-12 (p70), IL-13, IL-17A, IL-18, IP-10, Leptin, LIX, MCP-1, MIP-1α, MIP-2, RANTES, TNF-α, and VEGF.

### RNA extraction and sample quality assessment

RNA was isolated from hippocampus according to the manufacturer’s instructions with the RNeasy mini kit (Qiagen; Cat. No. 74104). Total eukaryotic RNA isolates were quantified using a fluorimetric RiboGreen assay. Total RNA integrity was assessed using capillary electrophoresis (e.g., Agilent BioAnalyzer 2100), generating an RNA Integrity Number (RIN). For samples to pass the initial QC step, they needed to quantify higher than 500 ng and have a RIN of 8 or greater.

### RNA library creation

Total RNA samples were converted to Illumina sequencing libraries using Illumina’s TruSeq RNA Sample Preparation Kit (Cat. # RS-122-2001 or RS-122-2002). In summary, the mRNA from a normalized input mass of total RNA was isolated using oligo-dT coated magnetic beads, fragmented and then reverse transcribed into cDNA. The cDNA was blunt-ended, A-tailed and indexed by ligating molecularly barcoded adaptors. Libraries were amplified using 15 cycles of PCR. The final library size distribution was validated using capillary electrophoresis and quantified using fluorimetry (PicoGreen) and Q-PCR. Indexed libraries were then normalized, pooled and size-selected to 320 bp (tight) using the PippinHT instrument.

### RNA cluster generation and sequencing

Pooled libraries were denatured and diluted to the appropriate clustering concentration (1.5pM for Mid-output and 1.8pM for High-output). Denatured and diluted libraries were loaded onto the NextSeq 550 cartridge and clustering occurs onboard the instrument. Once clustering was complete, sequencing immediately commenced using Illumina’s 2-color SBS chemistry. Upon completion of read 1, an index read 1 was performed depending on the library kit used. Finally, the library fragments were re-synthesized in reverse orientation and sequenced from the opposite end of the read 1 fragment to produce the paired end read 2.

### RNA primary analysis and de-multiplexing

Base call files for each cycle of sequencing were generated by Illumina Real Time Analysis (RTA) software. The base call files and run folders were streamed to servers maintained at the Minnesota Supercomputing Institute. Primary analysis and de-multiplexing were performed using Illumina’s bcl2fastq v2.20. The result of the bcl2fastq workflow was de-multiplexed FASTQ files for subsequent analysis.

### RNA-seq data generation and analysis

2 × 35 bp FastQ paired-end reads for 24 samples (*n* = 17.9 million average per sample) were trimmed using Trimmomatic (v 0.33) enabled with the optional “-q” option; 3 bp sliding-window trimming from 3′ end requiring minimum Q30. Quality control on raw sequence data for each sample was performed with FastQC. Read mapping was performed via Hisat2 (v2.1.0) using the rat genome (Rnor_6.0) as reference. Gene quantification was done via Feature Counts for raw read counts. Differentially expressed genes were identified using edgeR (negative binomial) feature in CLCGWB (Qiagen, Redwood City, CA) using raw read counts. We filtered the generated list based on a minimum 2× Absolute Fold Change and FDR corrected *p* < 0.05.

### Ingenuity pathway analysis

Differential gene counts were analyzed using Ingenuity Pathway Analysis (IPA)® software (QIAGEN, Redwood 185 City, CA, USA). Expression fold-changes calculated for the STZ-Sham vs. Sham and STZ-DFO vs. STZ-Sham group comparisons were uploaded to IPA. Analysis settings were specified as rat (species) and nervous system (tissue). Expression fold-change cutoff was predetermined and specified as >1.5 and <−1.5 which yielded approximately 1,000 genes for analysis in each comparison. *p*-values, *z*-scores, and the names of involved canonical disease pathways and functions were automatically calculated by the IPA software and directly exported. The activation state (“increased” or “decreased”) of each involved biological pathway is inferred by the software from the value of the corresponding *z*-score.

### IHC protocol and antibodies

Sections were sliced on a cryostat at 15 μm (Leica Biosystems, IL, USA). Slides underwent a heat-induced antigen retrieval processes and then incubated for an hour in a blocking buffer containing BSA (Sigma; IN, USA) and goat serum (Life Technologies; MA, USA) in a 1X PBS solution with Tween 20 (Sigma; IN, USA). Primary antibodies included Iba1 (Wako), CD68 (BioRad), GFAP (Abcam), NeuN (EMD Millipore), Caspase 3 (ThermoFisher), and Cleaved PARP1 (ThermoFisher). Slides were triple-labeled with combinations of Iba1, GFAP, and CD68, or NeuN, PARP, and Caspase3. All primary and secondary antibodies were diluted to 1:250 and 1:500, respectively, in blocking buffer. Primary antibodies were incubated overnight at 4°C. Secondary antibodies were incubated at room temperature and shielded from light for 1.5 h. Slides were cover-slipped using Fluoro Shield with DAPI (ThermoFisher).

### IHC image acquisition and analysis

Slides were imaged on an Olympus FV3000 confocal microscope using Fluoview software. The three antibodies on each slide had different absorbances and representative colors as follows: green (PARP), red (NeuN), and yellow (Caspase3), or green (Iba1), red (GFAP), and yellow (CD68). Images were taken using the Multi-area Time Lapse (MATL) feature at 20× and stitched together. The regions of interest (ROI) was the and hippocampus. Individual targetswere analyzed using Olympus cellSens Dimensions.

### Statistical analyses

For Morris water maze, repeated measures ANOVA with post-tests were used (Systat). ANOVAs with Fishers LSD post-tests were used for all other analyses as appropriate (GraphPad Prism).

## Results

### Behavioral testing

**Figure 2:**
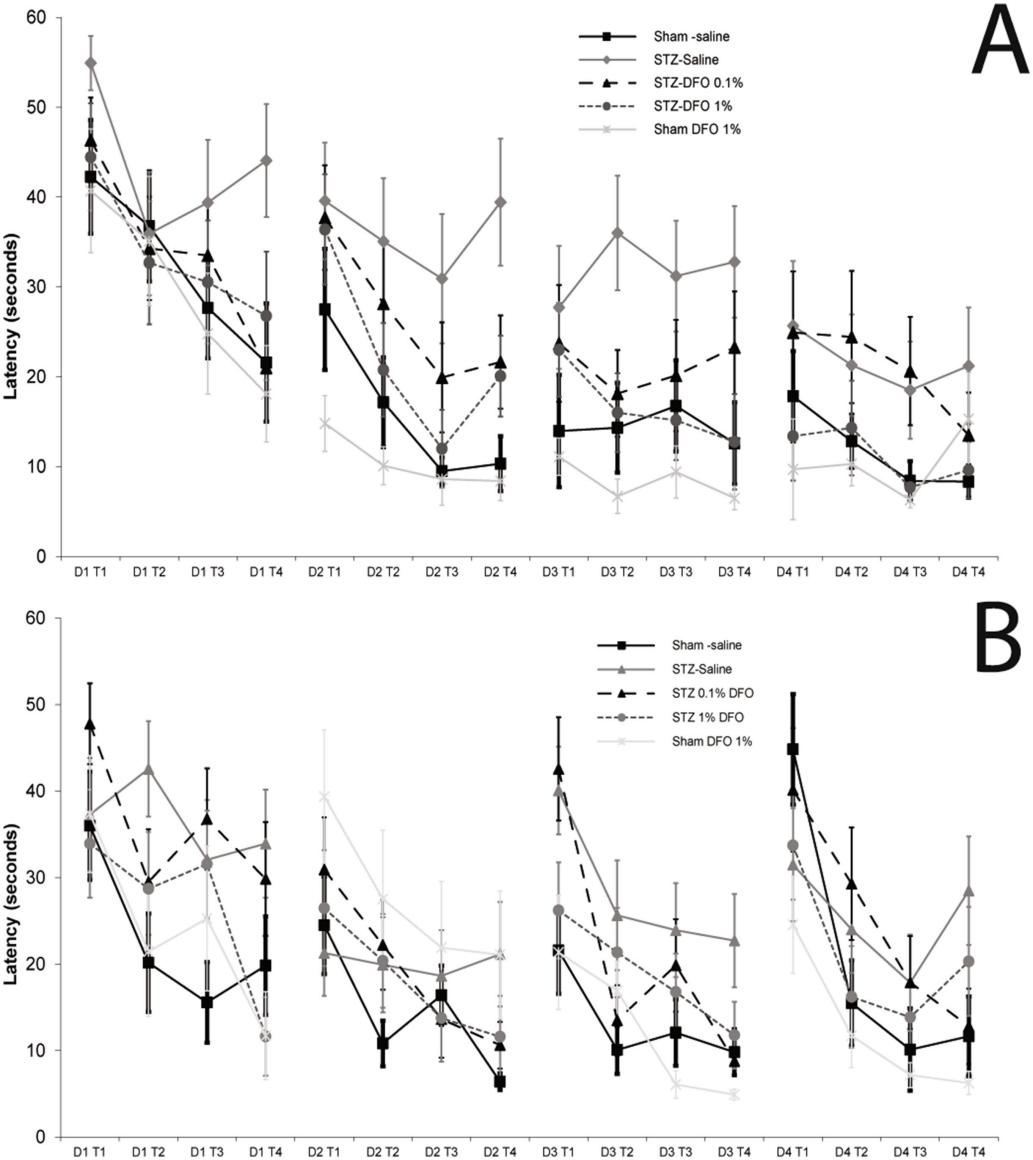
Morris water maze data with fixed platform **(A)** and moving platform **(B)** for sham and ICV-STZ model mice treated with intranasal saline or DFO. Concentrations of DFO were 0.1 and 1% solutions and mice were treated 5×/week for 5 weeks. Repeated measures ANOVA showed that there was a clear model effect of the ICV-STZ treatment, and that treatment with 1% IN DFO significantly decreased escape latency as compared to controls (*p* < 0.05), while treatment with 0.1% DFO did not.

There was a significant difference among all groups in fixed platform MWM performance (Figure 2A) as assessed by repeated measures ANOVA (*p* = 0.013). In the absence of the STZ model, IN DFO treatment alone did not improve performance, though there was a trend toward improvement in line with previous studies (Figure 2A; *p* = 0.15) (Fine et al., 2017). As expected, induction of the ICV STZ model resulted in significantly higher escape latencies compared to controls (*p* = 0.009), an indicator of memory loss in this rodent AD model. Treatment with 1% IN DFO reversed this model effect (*p* = 0.032), with 0.1% DFO treatment trending toward improvement (Figure 1A) but not reaching statistical significance (*p* = 0.23). For moving platform MWM, repeated measures ANOVA detected a significant group effect (*p* = 0.02; Figure 2B). The ICV-STZ model caused significantly increased escape latency compared to controls (*p* = 0.004) that was rescued with 1% DFO treatment (*p* = 0.042) but not 0.1% DFO treatment (*p* = 0.174, Figure 2B). Representative track plots for a single trial for a single rat from each treatment group can be seen in Supplementary Figure 1. There were no significant differences between any treatment groups for tapered balance beam, optomotor, or open field tests (data not shown).

### Neuroinflammation

**Figure 3:**
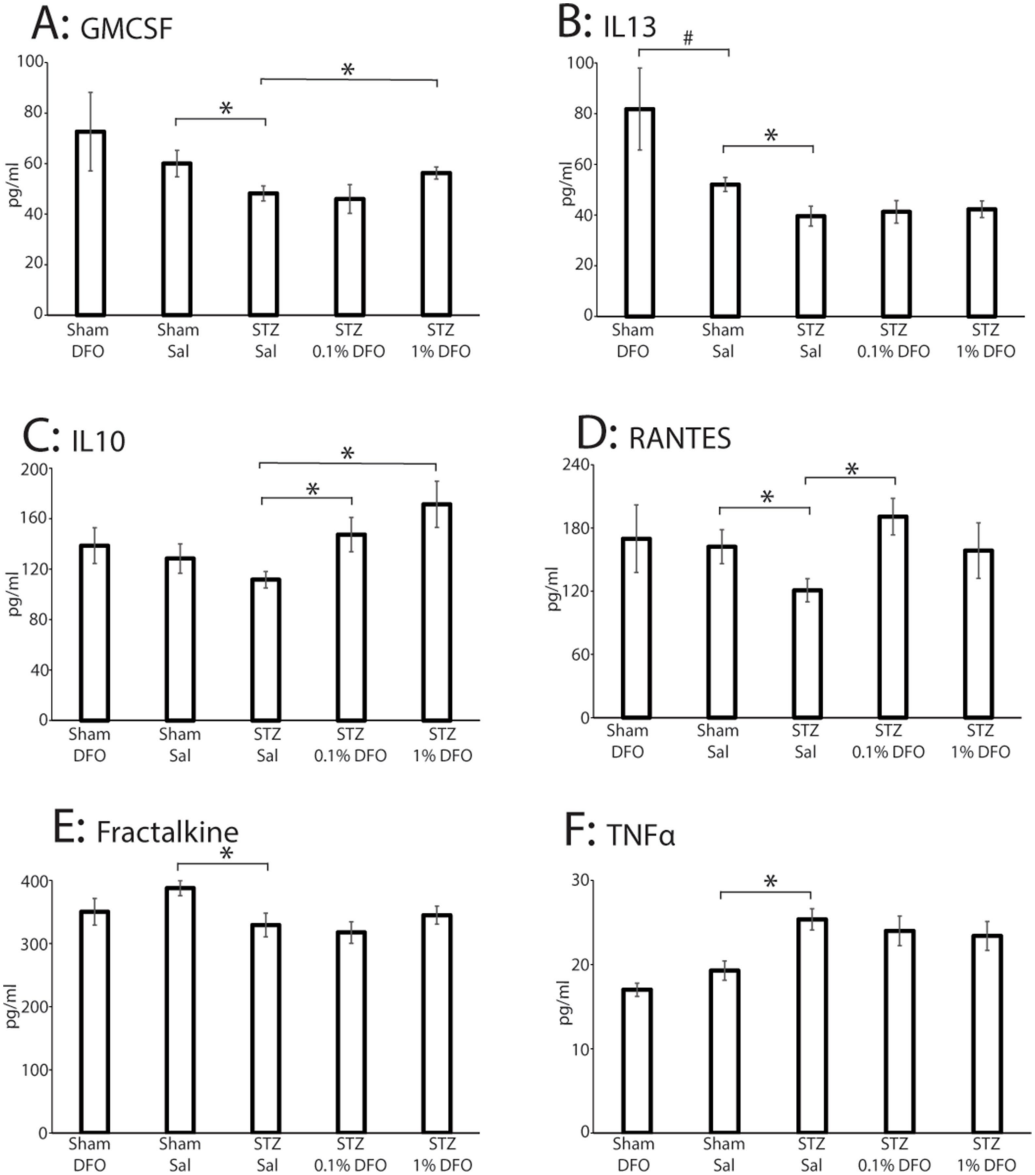
Mulitplex data from a panel of 27 inflammatory markers performed on hippocampal tissue from sham or ICV-STZ rats treated with intranasal saline or DFO. Data are only shown for the six markers with significant differences as measured with ANOVA with post-tests (*p < 0.05, #*p* < 0.1). **(A)** GMCSF, **(B)** IL13, **(C)** IL10, **(D)** RANTES, **(E)** Fractalkine, **(F)** TNFα. Sample sizes for each group are as follows: Sham-DFO = 7, Sham-Sal = 8, STZ-Sal = 12, STZ-0.1% DFO = 11, STZ-1% DFO = 11.

Out of 27 targets assessed by the Millipore Sigma™ cytokine array, 22 were detectable, and six were significantly changed (Figures 3A–F; histograms only shown for the six markers with significant statistical changes among treatment groups). STZ model induction resulted in significantly decreased levels of GM-CSF (*p* = 0.047), a neuroprotective cytokine (Schabitz et al., 2008; Kosloski et al., 2013), an effect reversed with 1% DFO treatment (*p* = 0.05, Figure 3A). STZ-treated animals likewise had significantly lower IL-13 (*p* = 0.035, Figure 3B), Fractalkine (*p* = 0.042, Figure 3E), and RANTES (*p* = 0.042, Figure 3D), cytokines that all play a significant anti-inflammatory or neuroprotective role (Quarta et al., 2020; Wang et al., 2021; Tripathy et al., 2010). Supporting these findings, STZ rodents had significantly elevated levels of TNF-α (*p* = 0.003, Figure 3F), a prominent pro-inflammatory cytokine (Habbas et al., 2015). In addition, 1% DFO treatment resulted in significantly increased IL-10 levels (*p* = 0.004, Figure 3C), a cytokine potentially involved in contributing to suppression of neuroinflammatory damage caused by induction of the ICV-STZ model, while RANTES was significantly increased in the 0.1% DFO group. Histograms for markers without significant changes are included in Supplementary Figure 2.

**Figure 4:**
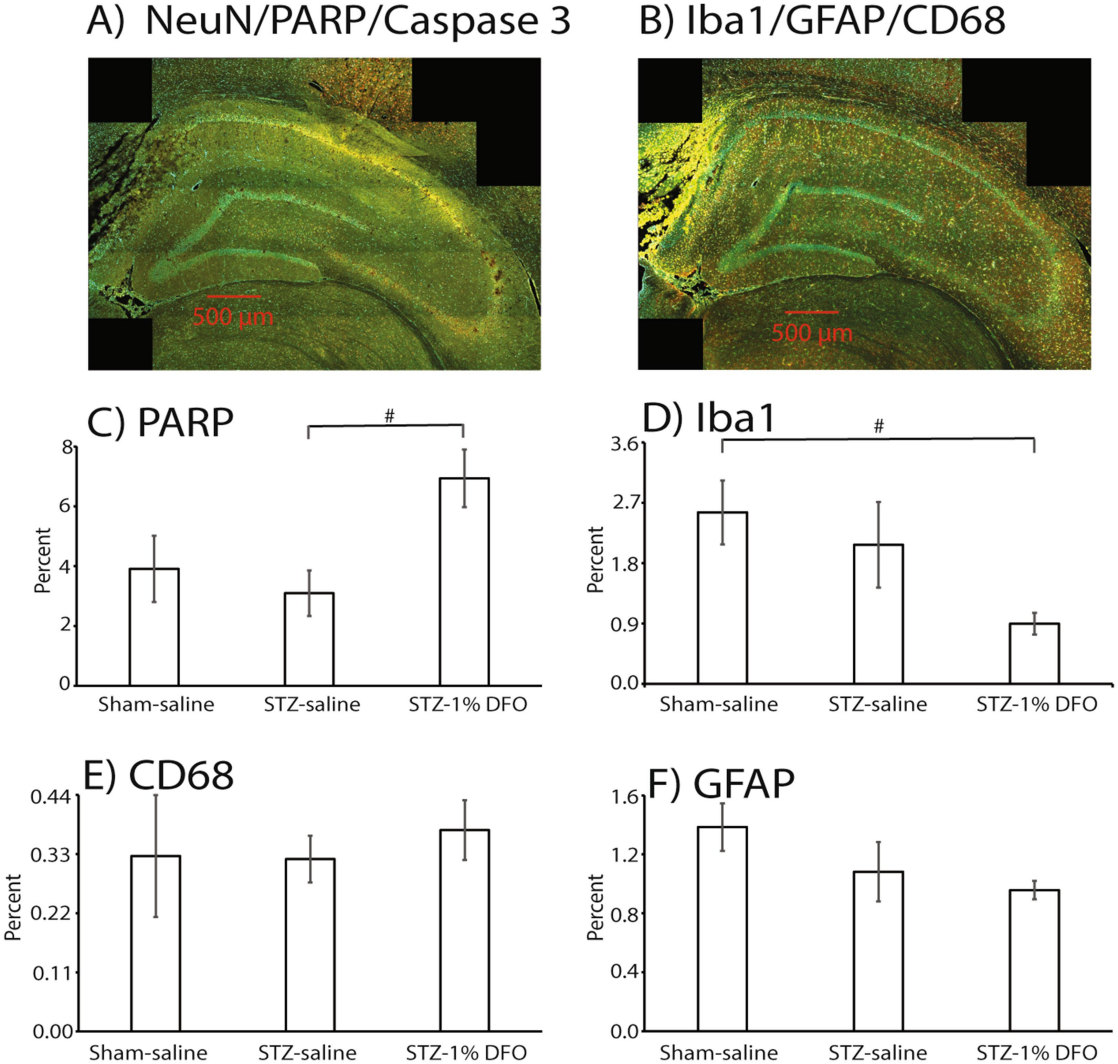
Immunohistochemical analyses of hippocampus in rats with either sham surgery or an ICV-STZ model of memory impairment at 14 days after model induction. A representative brain slice for imaging with PARP, NeuN, and Caspase 3 is in Panel **(A)**, while a slice with Iba1, CD68, and GFAP is included in panel **(B)**. The three antibodies on each slide had different absorbances and representative colors as follows: green (PARP), red (NeuN), and yellow (Caspase3), or green (Iba1), red (GFAP), and yellow (CD68). Both images are from an STZ rat treated with DFO (1%). Histograms are included and expressed as percent area fraction for PARP **(C)**, Iba1 **(D)**, CD68 **(E)**, and GFAP **(F)**. Sample size is 3/group, and # = *p* < 0.1. ANOVA with post-tests showed an insignificant trend toward a treatment effect with DFO for PARP and Iba1.

Neuroinflammation was also assessed within the hippocampus using three well-established immunohistochemical markers—Iba1 (Dos Santos et al., 2020) (staining for microglia, Figures 4B,D), CD68 (Majkutewicz et al., 2018) (staining for reactive microglia, Figures 4B,E), and GFAP (Chen et al., 2018) (staining for reactive astrocytes, Figures 4B,F). Quantities were expressed as percent area fraction. Although there was no clear change with CD68, there was a statistically insignificant trend for GFAP and Iba1 to decrease with DFO treatment (*p* = 0.077 for Iba1).

### Neuronal injury

Neuronal injury was assessed with IHC for three markers: NeuN (Chen et al., 2018) (a neuronal marker), activated Caspase-3 (Du et al., 2015) (a marker of cell apoptosis), and PARP (a marker of DNA damage and repair). There was a significant trend for DFO to increase levels of PARP in hippocampus (*p* = 0.08, Figures 4A,C), which may have been more significant with a bigger sample size. Differences among treatment groups were not detected for NeuN and Cas3 with ANOVA (data not shown).

### RNA-sequencing analysis

RNA-seq pathway analysis demonstrated that compared to controls, the STZ transcriptome featured significant involvement of the neuroinflammatory pathway (*p* = 6.82E−11). Upon more detailed functional analysis, a number of neurological disease-related pathways and networks were engaged in the STZ model (Table 1 and Supplementary Table 1). As expected, STZ reduced cell viability of neurons (*p* = 0.04) and specifically, hippocampal neurons (*p* = 0.005), and increased measures of neurodegeneration of brain (*p* = 0.05) and nervous system (*p* = 0.03). STZ caused activation of neuroglia (*p* = 0.01), proliferation of neuroglia (*p* = 0.007), and increased cell movement (*p* = 0.05), as well as more generalized proliferation of brain cells (*p* = 0.008) and CNS cells (*p* = 0.01). Likewise, STZ induced activation of neurons (*p* = 0.01) and increased quantities of neurotransmitters (*p* = 0.007) potentially suggestive of excitotoxicity.

**Table 1:** RNA-seq pathways modulated by STZ model induction.

Function/disease	*p*-value	*z*-score
Activation of neuroglia	0.0123	1.964
Activation of neurons	0.0104	0.728
Cell viability of hippocampal neurons	0.00555	−0.943
Cell viability of neurons	0.0365	−0.366
Degeneration of brain	0.0475	0.152
Degeneration of nervous system	0.0294	−0.152
Excitation of neurons	0.0222	0.277
Neurodegeneration	0.0294	−0.152
Proliferation of brain cells	0.00849	−0.555
Proliferation of central nervous system cells	0.0119	−0.128
Proliferation of neuroglia	0.00706	0.832
Quantity of neurotransmitter	0.00773	1.309

RNA-seq analysis also demonstrated that 1% DFO treatment reduced and reversed much of the engagement of these pathways (Table 2 and Supplementary Table 2). The transcriptome of DFO-treated animals compared to STZ-saline animals demonstrated decreased neuronal injury (*p* = 0.001), nervous system injury (*p* = 0.01), neurodegeneration (*p* = 0.003), cell degeneration (*p* = 0.01), neuronal degeneration (*p* = 0.03), neuron damage (*p* = 0.005), brain damage (*p* = 0.03), and nervous system damage (*p* = 0.02). In addition, DFO treatment resulted in robustly increased networks associated with neuronal viability (*p* = 0.03), hippocampal neuron (*p* = 0.03) and cell viability (*p* = 0.007), nervous system differentiation (*p* = 0.03), CNS differentiation (*p* = 0.03), and CNS development (*p* = 0.007).

**Table 2:** RNA-seq pathways modulated by DFO treatment within the STZ model.

Function/disease	*p*-value	*z*-score
Injury of neurons	0.00164	−1.924
Damage of hippocampal neurons	0.0025	
Degeneration of nervous system	0.00277	−1.614
Neurodegeneration	0.00277	−1.765
Chemotaxis	0.00306	2
Proliferation of central nervous system cells	0.00333	1.213
Damage of neurons	0.00586	−1.051
Development of central nervous system	0.0066	2.145
Cell viability of hippocampal cells	0.00724	1.457
Degeneration of cells	0.0111	−1.294
Injury of nervous system	0.0122	−2.366
Damage of nervous system	0.0156	−1.623
Proliferation of brain cells	0.0255	1.664
Cell viability of hippocampal neurons	0.0286	1.176
Degeneration of neurons	0.0295	−1.709
Brain damage	0.0316	−2.191
Differentiation of nervous system	0.0319	1.961
Cell viability of neurons	0.0327	2.378

## Discussion

Intranasal (IN) deferoxamine (DFO) has emerged as an increasingly promising therapeutic for neurodegenerative diseases, especially AD (Kosyakovsky et al., 2021; Farr and Xiong, 2021). It has shown robust, widespread efficacy between multiple research groups and across multiple preclinical models of disease, with evidence that it reaches the brain predominantly via the olfactory route (Kosyakovsky et al., 2019). As we demonstrate herein, a low dose of 1% IN DFO rescues cognitive deficits in the ICV-STZ rat model, reversing hippocampal neuronal loss, neuronal apoptosis, and multiple markers of neuroinflammation. The behavioral data in this study demonstrate that a daily dose of 1% IN DFO is beneficial for memory retention in the ICV-STZ model of sporadic AD, while the 0.1% IN DFO did not have the same effect. This data supports Fine et al. (2017), in which IN DFO given at 10% was beneficial in the same model, and sets a clear dose response with a lower limit of benefit. The 1% DFO dose did not have an effect on vision, motor coordination, or anxiety as measured by optomotor, tapered balance beam, or open field tests, respectively. This differs from the data in Fine et al. (2017) for the tapered balance beam, in which the 10% DFO dose did show benefit, supporting evidence for a dose–response for motor coordination.

The results of the cytokine array multiplex data confirm previous studies that have suggested that STZ-induced hippocampal dysfunction involves a pro-neuroinflammatory milieu (Grieb, 2016; Dos Santos et al., 2020), and that DFO acts to counter neuroinflammation. The ICV-STZ model decreased anti-inflammatory cytokines including FKN (fractalkine), IL13 (interleukin 13), RANTES (Regulated on Activation, Normal T cell Expressed and Secreted), and GMCSF (granulocyte macrophage colony stimulating factor) while promoting the pro-inflammatory cytokine, TNFα (tumor necrosis factor α). Intranasal DFO promoted the expression of IL10 (interleukin 10) and GM-CSF, which are both anti-inflammatory. Some of these results are supported in other studies of both ICV-STZ rats and other models of Alzheimer’s disease. Kumar et al. (2017) showed that the overexpression of TNF-α is caused by the overactivation of microglia and astrocytes due to STZ administration. IL13 causes a cascade of signals that downregulates the production of proinflammatory cytokines such as TNF-α (Cuneo and Autieri, 2009), showed benefit in an amyloid mouse model of AD (Kawahara et al., 2012). In an APP/PS1 mouse model, GM-CSF expression decreased amyloid deposition, significantly improved cognitive functioning, decreased cell death, and increased neurogenesis (Boyd et al., 2010). In an STZ-diabetic mouse model, GM-CSF reduced inflammation and promoted healing of STZ-induced fractures (Krakowski et al., 2002). FKN signaling offers neuroprotection in AD pathology by decreasing cell death, inflammation, and tau accumulation and by increasing synaptic plasticity (Finneran and Nash, 2019; Sheridan et al., 2014). Intranasal IL-10 administration in a mouse model of depression resulted in improvement of learning and memory (Worthen et al., 2020). Also, in an STZ-induced diabetic mouse model, IL-10 expression aided in the repair of damage caused by the model and decreased the overall inflammatory response (Cui et al., 2020). Thus, cytokines play an important role in the ICV-STZ model, and DFO acts to counter neuroinflammation.

RNA-sequencing demonstrates that STZ administration engages transcriptomic networks involved in neuronal death and dysfunction accompanied by neuroinflammatory change. These effects were significantly reduced by the neuroprotective transcriptional phenotype associated with DFO treatment. The power of transcriptomics stems from this method’s ability to synthesize a vast array of seemingly disparate gene expression data into functional pathways and modules with known correlation to function and disease (Pairo-Castineira et al., 2021; Litvinukova et al., 2020; Grant et al., 2021). As expected, transcriptomic analysis confirmed that the ICV-STZ model creates a cellular environment for increased neuroinflammation and neuronal death compared to normal animals. Conversely, DFO treatment was associated with decreased activation of inflammatory pathways and promotion of pathways involved in cellular survival. These findings support our understanding of both the mechanisms of STZ as a model of AD as well as the therapeutic effects of DFO in this setting (Kosyakovsky et al., 2021; Grieb, 2016).

The IHC data give interesting insight into neuronal injury and inflammation in the hippocampus. The primary mechanism of the STZ model centers on hippocampal injury (Grieb, 2016), with ensuing neuroinflammation and cognitive deficits in ICV STZ rodents. Likewise, the mechanisms of DFO treatment with translational relevance to neurodegenerative disease center on neuroprotection and reducing neuronal injury, pathways underpinning its widespread efficacy across preclinical models of neurological disease. Although four rats were initially used for the two STZ groups, mortality in each group before euthanasia led to a sample size of only three in each group for analysis making it challenging to pick up significant differences with ANOVA. There were still some interesting trends, most notably with PARP. PARP is a nuclear protein tightly linked to the cellular response to oxidative stress and hypoxia (Wang et al., 2019; Abeti and Duchen, 2012; Marti et al., 2020). Intranasal DFO has been shown to strengthen the post-hypoxia and oxidative stress response (Fine et al., 2012; Fine et al., 2015), one of the main mechanisms by which it is believed to be efficacious in neurodegenerative and cerebrovascular disease (Kosyakovsky et al., 2021). In part, this effect has been shown to stem from its impact on PARP activation and engagement of subsequent cellular pathways (Martinez-Romero et al., 2008; Canuelo et al., 2012). In this study, we found that 1% DFO treatment was significantly associated with PARP activation. This finding strengthens our understanding of DFO’s effect on the oxidative milieu, with PARP-modulated hypoxic response induction perhaps underpinning hippocampal neuroprotection (Canuelo et al., 2012; Kosyakovsky et al., 2021).

Regarding the translation of IN DFO to clinical trials, our group recommends the following dosage range based on the effective and well-tolerated rat dosing concentration range of 1–10% DFO with a volume of 30 μL per rat, described both in this paper and several others (Fine et al., 2012; Fine et al., 2015; Fine et al., 2017; Fine et al., 2020). First, when scaling up to human doses it is important to note that mg/kg dosing should not be used since intranasal drugs administered to the upper third of the nasal cavity travel directly from the nose to the brain extracellularly along the olfactory and trigeminal neural pathways. They do not need to first distribute throughout the bloodstream and body and subsequently penetrate the blood–brain barrier to reach their therapeutic targets in the brain. For example, intranasal insulin reaches the cerebrospinal fluid within 10 min without altering the blood levels of insulin or glucose (Born et al., 2002) and has been shown to safely improve memory in healthy adults (160 IU/day) (Benedict et al., 2004), people with type-2-diabetes (40 IU/day) (Novak et al., 2022), and mild cognitive impairment or Alzheimer’s disease (20 or 40 IU/day) (Reger et al., 2006). Intranasal DFO results in brain concentrations that are significantly higher than blood concentrations achieved in the same animals (Kosyakovsky et al., 2019). Second, we recommend initial clinical trials maintain the same effective and well tolerated rat dosing concentration range since exposure to the olfactory and trigeminal cranial nerve endings in the nasal mucosa will be similar in humans. Third, we recommend increasing the volume administered from 30 μL in the rat to 200 μL (100 μL per nostril) in humans since the nasal cavity volumes and nasal mucosa areas are considerably larger in humans. Also, a 100 μL volume per nostril is a well-tested nasal spray volume in humans. For the purpose of successful IN delivery to the olfactory and trigeminal nerve endings, we recommend using a nasal spray device that is capable of reaching the olfactory epithelium at the roof of the nasal cavity. Beginning with 1% DFO in humans and increasing the concentration from there to 10% to assess safety and efficacy is suggested.

Clinical trials of iron chelators for neurodegenerative diseases started many years ago and are still underway. As mentioned previously, an initial clinical trial of DFO for Alzheimer’s disease was published in 1991 and showed promise as a treatment, but was discontinued due to systemic side-effects (Crapper McLachlan et al., 1991). Very recently, a clinical trial of another metal chelator approved for iron overload, deferiprone, was conducted (Ayton et al., 2024). Because it can be delivered orally, it was tested in a clinical trial for Alzheimer’ disease but was correlated with negative outcomes for AD. Although this is not promising for the use of metal chelators as a treatment for AD, it should be noted that there are several differences from DFO including the fact that it is a different drug, delivery was oral, there were systemic side-effects, and the dose was relatively high. In preparation for clinical trials with IN DFO, safety and toxicity studies performed in our lab under the principles of good laboratory practice have shown that there is minimal detrimental effects of IN DFO in rats (Hanson et al., unpublished), and it still seems worthy of pursuit as a treatment.

The failure of many AD therapeutics has been attributed in part to the inadequacy of rodent models (Mullane and Williams, 2019). Ultimately, we do not believe that the ICV-STZ model, which we characterized herein using immunohistochemistry, a cytokine panel, and a novel RNA-seq investigation, captures human AD better than alternate models. However, it offers an alternative to amyloid models with a neurodegenerative and neuroinflammatory milieu in which to test therapeutics. Several of these mechanisms are included in the cascade of ferroptosis (Stockwell, 2022). In this study, we provide the first transcriptomic analysis of IN DFO treatment that confirm its widespread neuroprotective and pro-survival impact on gene networks *in vivo*. These findings will inform dose regimens for future clinical trials of IN DFO and cast additional light on its multimodal mechanisms.

## Data Availability

The RNA sequencing data presented in this study can be found in online repositories. The names of the repository/repositories and accession number(s) can be found at: https://www.ncbi.nlm.nih.gov/geo/, GSM8630177; https://www.ncbi.nlm.nih.gov/geo/, GSM8630178; https://www.ncbi.nlm.nih.gov/geo/, GSM8630179; https://www.ncbi.nlm.nih.gov/geo/, GSM8630180; https://www.ncbi.nlm.nih.gov/geo/, GSM8630181; https://www.ncbi.nlm.nih.gov/geo/, GSM8630182; https://www.ncbi.nlm.nih.gov/geo/, GSM8630183; https://www.ncbi.nlm.nih.gov/geo/, GSM8630184; https://www.ncbi.nlm.nih.gov/geo/, GSM8630185. Additionally, the raw data supporting the conclusions of this article will be made available by the authors, without undue reservation, to any qualified researcher.
