# Supplementary material for: Low-dose intranasal deferoxamine modulates memory, neuroinflammation, and the neuronal transcriptome in the streptozotocin rodent model of Alzheimer’s disease
Source: Front Neurosci. 2025 Jan 13;18:1528374. doi: 10.3389/fnins.2024.1528374 (PMC11770042; doi:10.3389/fnins.2024.1528374)
Supplement: Supplementary file 1 [file Data_Sheet_1.pdf]

Supplementary Material

Supplementary Table 1. RNA-seq molecules affected by STZ model induction

Function/Disease	Molecules
Activation of neuroglia	Cdkn1c,CX3CR1,IL1B,P2rx7
Activation of neurons	CARTPT,IL1B,IL6,OXT,POMC,RET
Cell viability of hippocampal neurons	IGF1,IGF2,IL1B,PTGDR2,TRIB3
Cell viability of neurons	BCL2,CDK1,CNTF,CTF1,HSPB1,IGF1,IGF2,IL1B,NTF4,PPP1R17,PTGDR2,REG3A,TRIB3
Degeneration of brain	CNTF,IGF1,IL6,TRIB3
Degeneration of nervous system	CNTF,IGF1,IL1B,IL6,NTF4,OSMR,SERPINE1,TRIB3
Excitation of neurons	FAAH,GCK,HSD11B2,KCNS1,SLC2A3
Neurodegeneration	CNTF,IGF1,IL1B,IL6,NTF4,OSMR,SERPINE1,TRIB3
Proliferation of brain cells	CALCB,GRN,IGF1,IL1A,IL1B,IL6,MELK,TGFB1
Proliferation of central nervous system cells	A2M,CALCB,GRN,IGF1,IL1A,IL1B,IL6,MELK,TGFB1
Proliferation of neuroglia	AREG,ASCL2,CXCL2,FAS,FN1,GFAP,IGF1,IL1B,IL6,LEPR,LYN,TGFB1,VIM
Quantity of neurotransmitter	GCH1,IL1B,NTS,SLC6A4,TLR4

Supplementary Table 2. RNA-seq molecules modulated by DFO treatment within the STZ model

Function/Disease	Molecules
Injury of neurons	ANGPT2,FGF2,IGF1,IGF2
Damage of hippocampal neurons	FGF2,IGF1,IGF2
Degeneration of nervous system	C6,F2,FGF2,IGF1,IL1RN,IL6,NTF4,OSMR,SERPINE1,TRIB3
Neurodegeneration	C6,F2,FGF2,IGF1,IL1RN,IL6,NTF4,OSMR,SERPINE1,TRIB3
Chemotaxis	A2M,LRP2,NTN1,SEMA3F
Proliferation of central nervous system cells	A2M,EGF,ERBB2,F2,FGF2,IGF1,IL1RN,IL6,INHBA,MELK
Damage of neurons	ANGPT2,DAO,FGF2,IGF1,IGF2,IL6
Development of central nervous system	AGTR2,AK8,ALDH1A2,ALDH1A3,AQP1,B3GNT5,BMP5,BMP7,CERS1,COL2A1,COL3A1,CYP26A1,DRD2,DUSP10,EFNA5,EN1,EN2,ENPP2,EOMES,EPOR,ERBB2,FGF2,FOXC1,FOXP2,FZD3,GHRHR,GRHL2,GSX1,GSX2,HES3,HPRT1,IGF1,LHX1,LMX1A,LRP2,MDK,MET,mir-124,mir-219,MSX1,NEUROG2,NGFR,NIN,NKX2-1,NMUR2,PAX8,PITX3,POU1F1,PPARG,SLC1A2,SLC6A3,SPAG6,SPHK1,THBS2,TNFRSF1B,TRPV4,VAX1,VTN,WNT5A,ZIC1,ZIC2,ZIC5
Cell viability of hippocampal cells	FGF2,IGF1,IGF2,PTGDR2,TRIB3
Degeneration of cells	C6,F2,FGF2,IL1RN,NTF4,OSMR,SERPINE1,TRIB3
Injury of nervous system	ANGPT2,FGF2,IGF1,IGF2,IL1RN,IL6
Damage of nervous system	ANGPT2,DAO,FGF2,IGF1,IGF2,IL1RN,IL6
Proliferation of brain cells	EGF,FGF2,IGF1,IL1RN,IL6,INHBA,MELK
Cell viability of hippocampal neurons	IGF1,IGF2,PTGDR2,TRIB3
Degeneration of neurons	F2,FGF2,IL1RN,NTF4,OSMR,SERPINE1,TRIB3
Brain damage	FGF2,IGF1,IGF2,IL1RN,IL6
Differentiation of nervous system	ADGRG6,CBLN1,DUSP10,ENPP2,ERBB2,FGF2,GPR157,GSX2,IGF1,INHBA,LMX1A,MCOLN3,MDK,mir-219,NEUROD4,NGFR,NKX2-1,NTF4,PITX3,POU1F1,PPARG,SFRP1,TNFRSF1B,USH2A,VEGFD,VTN,WNT5A
Cell viability of neurons	CTF1,FGF2,GDF15,IGF1,IGF2,IL11,NGFR,NTF4,PPP1R17,PTGDR2,REG3A,TGM2,TRIB3

Supplemental Figure 1. Representative track plots from fixed platform MWM (from various trials on Day 4) were acquired for a single trial from a rat from each treatment group. Data were acquired with Noldus Ethovision tracking software.

A. Sham Saline (Rat #112, Trial 1, Escape Latency=4 sec)

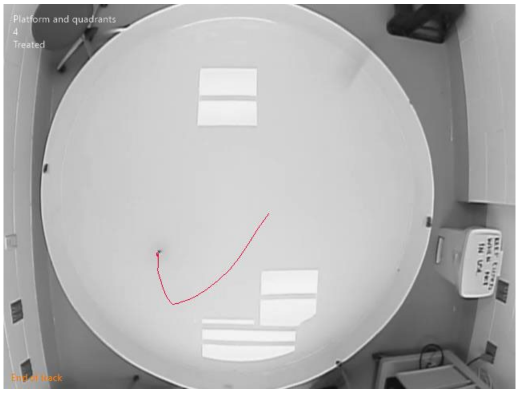

B: STZ-Saline (Rat #119, Trial 4, Escape Latency=60 sec)

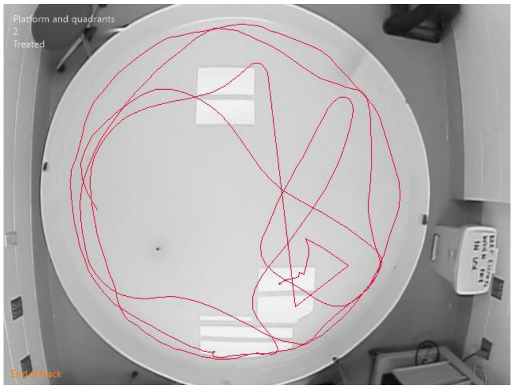

C. STZ-DFO (0.1%) (Rat #115, Trial 2, Escape Latency-6 sec)

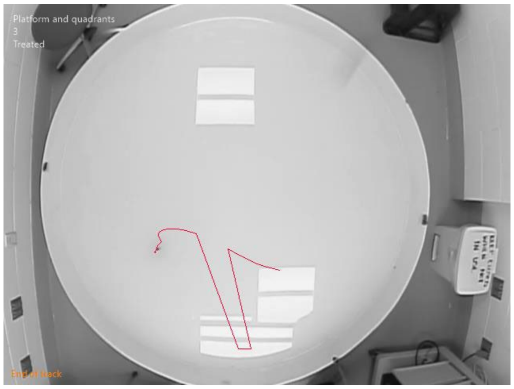

D: STZ-DFO (1%) (Rat #128, Trial 2, Escape Latency-10 sec)

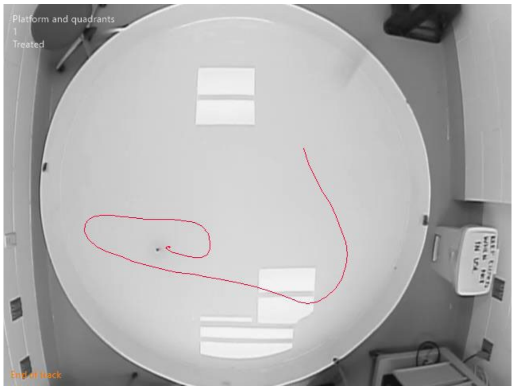

E: Sham- DFO (1%) (Rat #7, Trial 4, Escape Latency=16 sec)

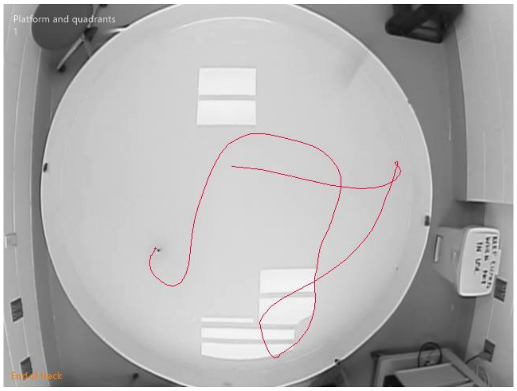

Supplemental Figure 2. Histograms of inflammatory markers that were detectable but did not have statistically significant changes between treatment groups. All are labelled by analyte, and the y-axis is pg/ml.

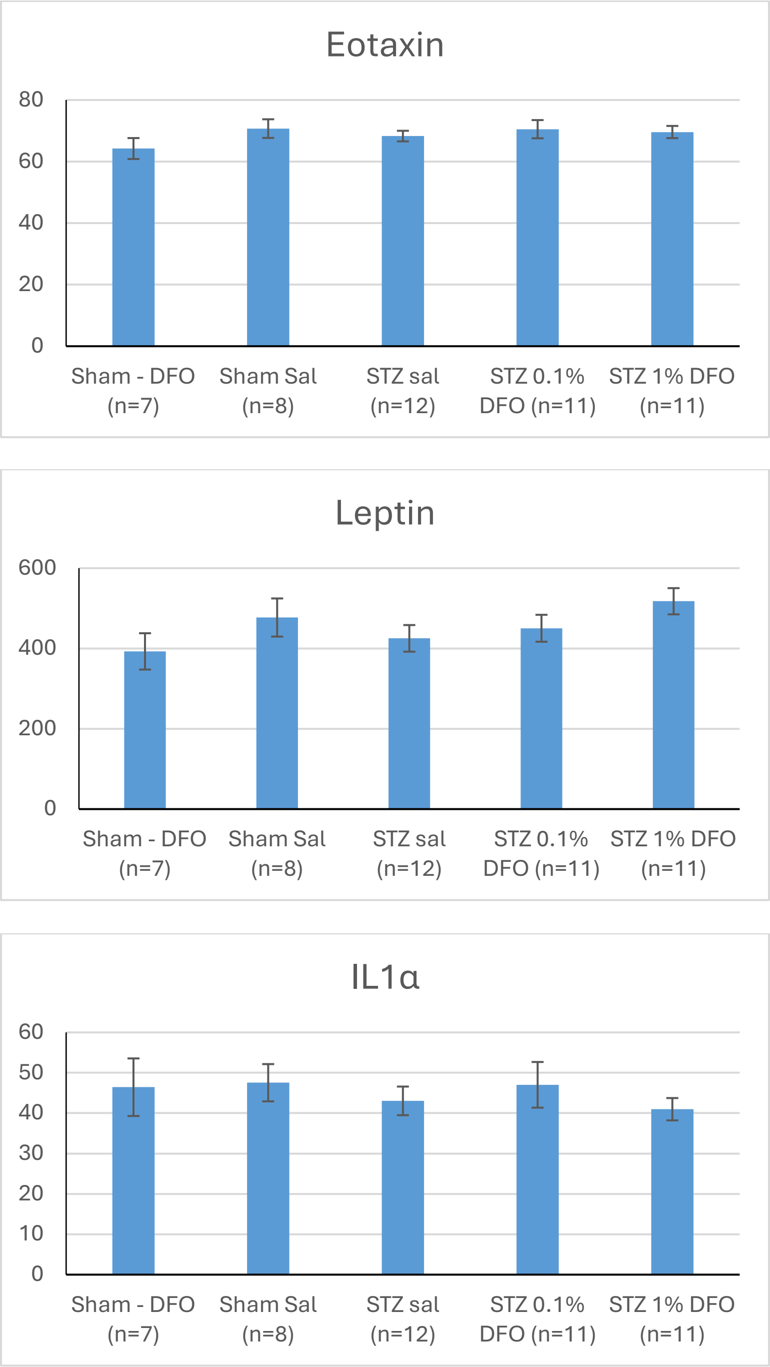

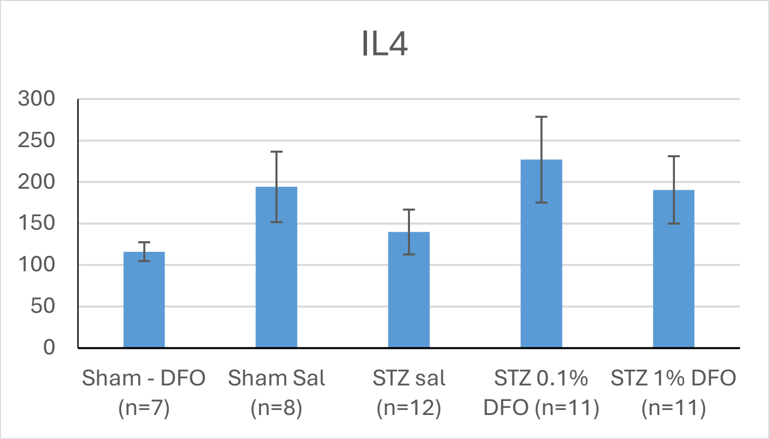
IL4
300
250
200
150
100
50
0
Sham - DFO (n=7)
Sham Sal (n=8)
STZ sal (n=12)
STZ 0.1% DFO (n=11)
STZ 1% DFO (n=11)

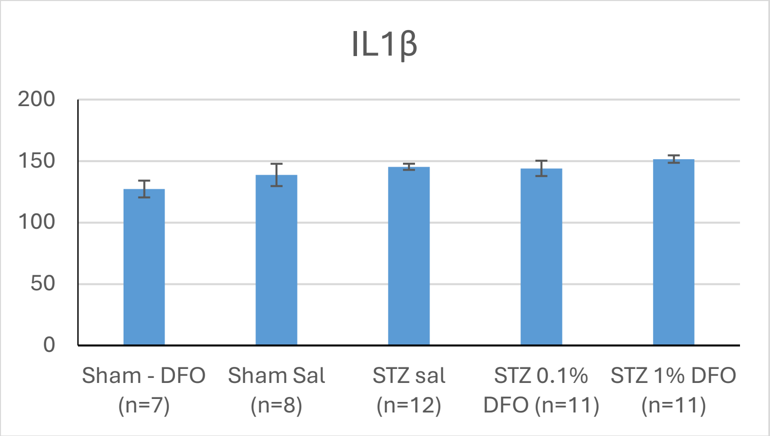
IL1β
200
150
100
50
0
Sham - DFO (n=7)
Sham Sal (n=8)
STZ sal (n=12)
STZ 0.1% DFO (n=11)
STZ 1% DFO (n=11)

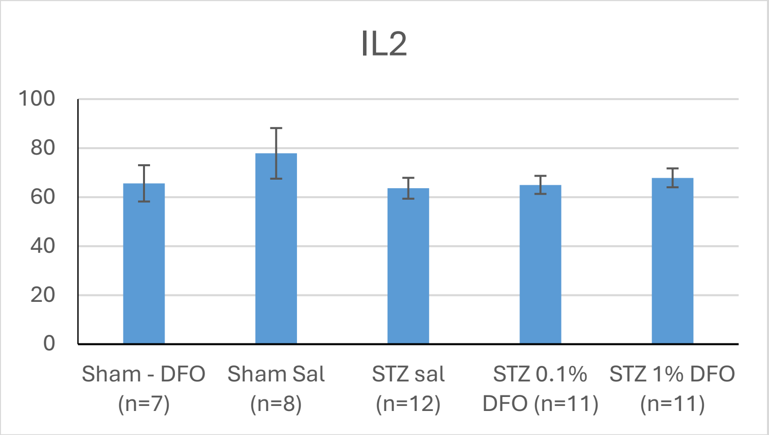
IL2
100
80
60
40
20
0
Sham - DFO (n=7)
Sham Sal (n=8)
STZ sal (n=12)
STZ 0.1% DFO (n=11)
STZ 1% DFO (n=11)

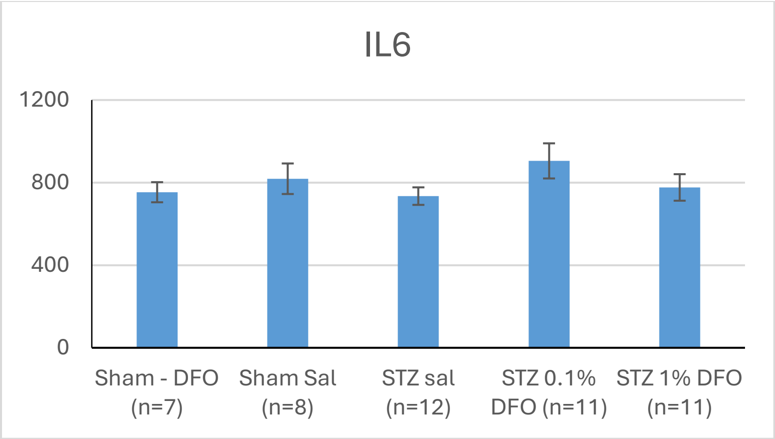
IL6
1200
800
400
0
Sham - DFO (n=7)
Sham Sal (n=8)
STZ sal (n=12)
STZ 0.1% DFO (n=11)
STZ 1% DFO (n=11)

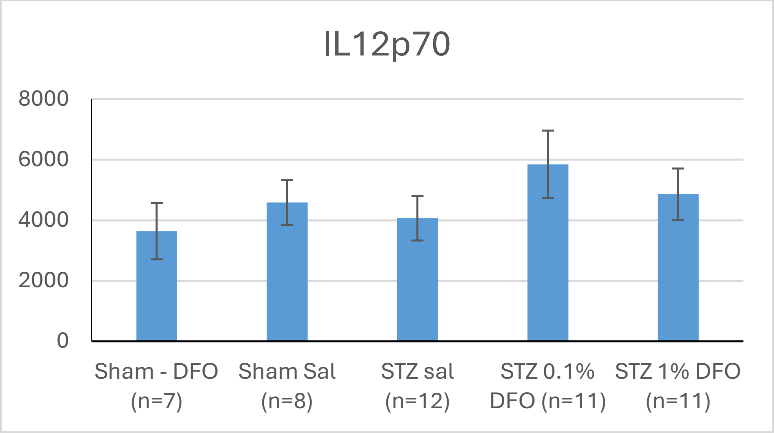
IL12p70
8000
6000
4000
2000
0
Sham - DFO (n=7)
Sham Sal (n=8)
STZ sal (n=12)
STZ 0.1% DFO (n=11)
STZ 1% DFO (n=11)

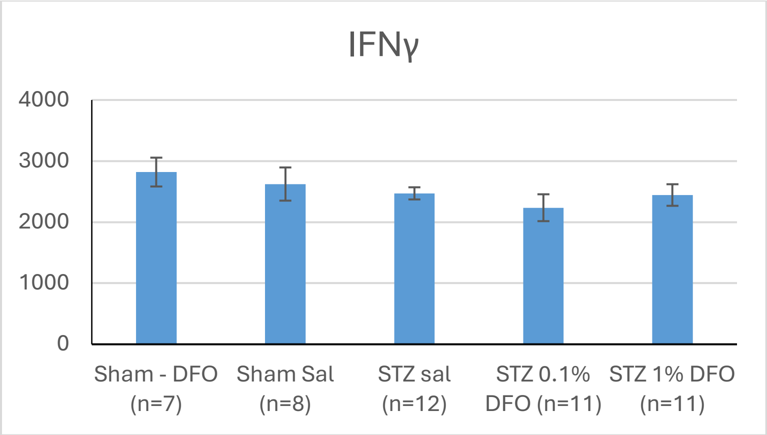
IFNγ
4000
3000
2000
1000
0
Sham - DFO (n=7)
Sham Sal (n=8)
STZ sal (n=12)
STZ 0.1% DFO (n=11)
STZ 1% DFO (n=11)

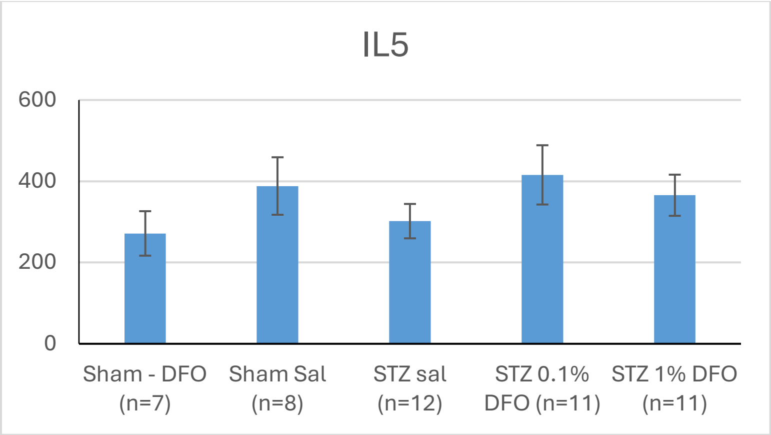
IL5
600
400
200
0
Sham - DFO (n=7)
Sham Sal (n=8)
STZ sal (n=12)
STZ 0.1% DFO (n=11)
STZ 1% DFO (n=11)

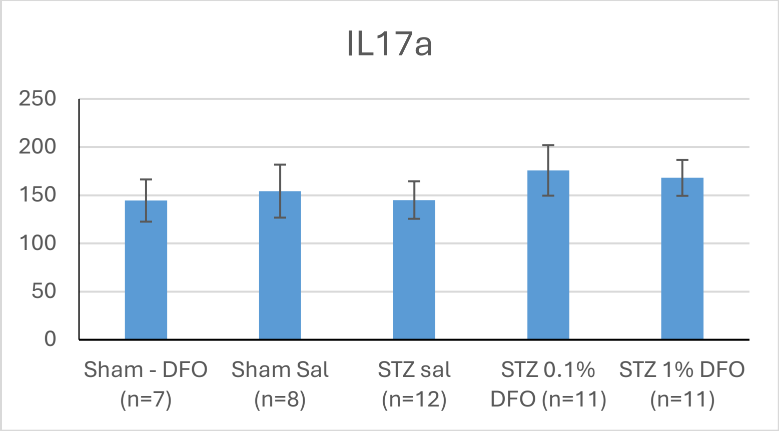
IL17a
250
200
150
100
50
0
Sham - DFO (n=7)
Sham Sal (n=8)
STZ sal (n=12)
STZ 0.1% DFO (n=11)
STZ 1% DFO (n=11)

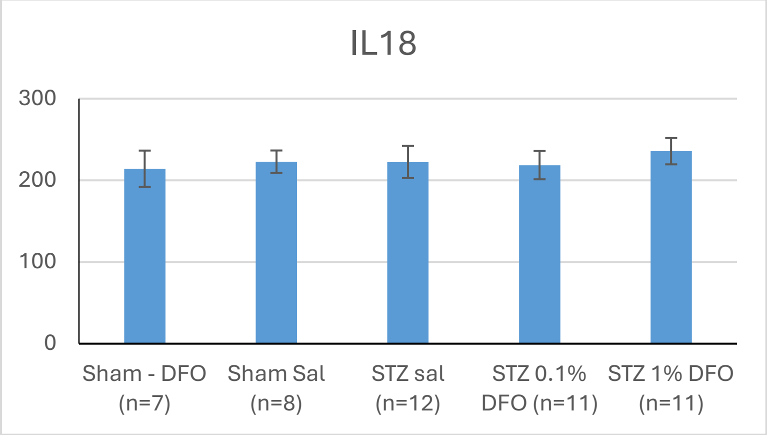
IL18
300
200
100
0
Sham - DFO (n=7)
Sham Sal (n=8)
STZ sal (n=12)
STZ 0.1% DFO (n=11)
STZ 1% DFO (n=11)

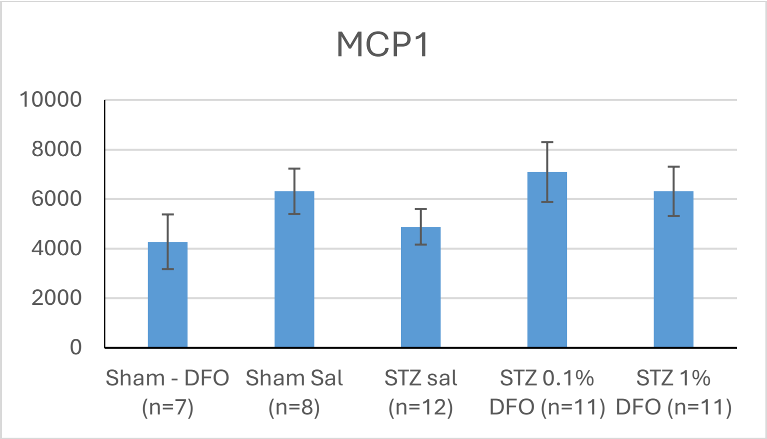
MCP1
10000
8000
6000
4000
2000
0
Sham - DFO (n=7)
Sham Sal (n=8)
STZ sal (n=12)
STZ 0.1% DFO (n=11)
STZ 1% DFO (n=11)

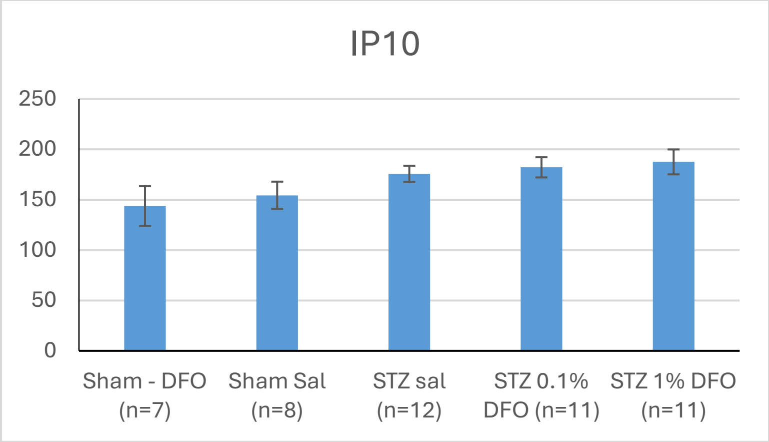
IP10
250
200
150
100
50
0
Sham - DFO (n=7)
Sham Sal (n=8)
STZ sal (n=12)
STZ 0.1% DFO (n=11)
STZ 1% DFO (n=11)

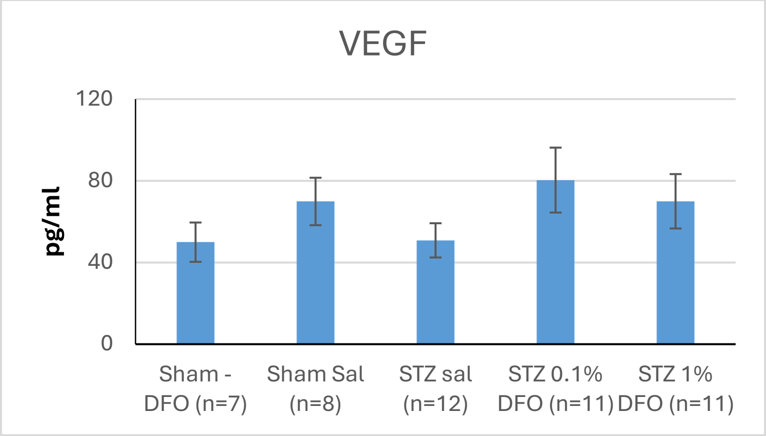
VEGF
pg/ml
120
80
40
0
Sham - DFO (n=7)
Sham Sal (n=8)
STZ sal (n=12)
STZ 0.1% DFO (n=11)
STZ 1% DFO (n=11)

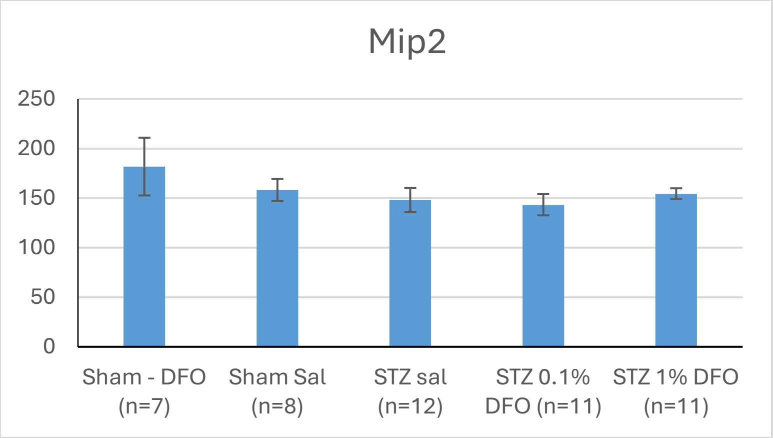
Mip2
250
200
150
100
50
0
Sham - DFO (n=7)
Sham Sal (n=8)
STZ sal (n=12)
STZ 0.1% DFO (n=11)
STZ 1% DFO (n=11)
